# Triptolide mitigates the inhibition of osteogenesis induced by TNF-α in human periodontal ligament stem cells via the p-IκBα/NF-κB signaling pathway: an in-vitro study

**DOI:** 10.1186/s12906-024-04408-2

**Published:** 2024-03-06

**Authors:** Hao Chen, Lina Zhang, Simeng Du, Daiwei Yang, Xiaobin Cui, Huadong Zhao, Jun Zhang

**Affiliations:** 1 Department of Orthodontics, School and Hospital of Stomatology, Cheeloo College of Medicine, Shandong Key Laboratory of Oral Tissue Regeneration & Shandong Engineering Laboratory for Dental Materials and Oral Tissue Regeneration & Shandong Provincial Clinical Research Center for Oral Diseases, Shandong University, No.44-1 Wenhua Road West, Jinan, 250012 Shandong Province China; 2 Science and Technology Innovation Committee of Shenzhen Municipality, Shenzhen Research Institute of Shandong University, A301 Virtual University Park in South District of Shenzhen, Shenzhen, 518063 Guangdong Province China; 3 Department of Orthodontics, Liaocheng People’s Hospital, Liaocheng, 252000 Shandong Province China

**Keywords:** Triptolide, hPDLSCs, Osteogenesis, Periodontitis, NF-κB

## Abstract

**Background:**

Triptolide is a widely utilized natural anti-inflammatory drug in clinical practice. Aim of this study was to evaluate effects of triptolide on hPDLSCs osteogenesis in an inflammatory setting and to investigate underlying mechanisms.

**Methods:**

Using the tissue block method to obtain hPDLSCs from extracted premolar or third molar. Flow cytometry, osteogenic and adipogenic induction were carried out in order to characterise the features of the cells acquired. hPDLSC proliferative activity was assessed by CCK-8 assay to determine the effect of TNF-α and/or triptolide. The impact of triptolide on the osteogenic differentiation of hPDLSCs was investigated by ALP staining and quantification. Osteogenesis-associated genes and proteins expression level were assessed through PCR and Western blotting assay. Finally, BAY-117,082 was used to study the NF-κB pathway.

**Results:**

In the group treated with TNF-α, there was an elevation in inflammation levels while osteogenic ability and the expression of both osteogenesis-associated genes and proteins decreased. In the group co-treated with TNF-α and triptolide, inflammation levels were reduced and osteogenic ability as well as the expression of both osteogenesis-associated genes and proteins were enhanced. At the end of the experiment, both triptolide and BAY-117,082 exerted similar inhibitory effects on the NF-κB pathway.

**Conclusion:**

The osteogenic inhibition of hPDLSCs by TNF-α can be alleviated through triptolide, with the involvement of the p-IκBα/NF-κB pathway in this mechanism.

**Supplementary Information:**

The online version contains supplementary material available at 10.1186/s12906-024-04408-2.

## Background

Periodontitis has a high incidence worldwide, with a significant impact on the health of periodontal supporting tissues [1, 2]. It is typically characterized by the deep periodontal pockets (≥ 4 mm), loss of attachment and resorption of alveolar bone. As the disease progresses towards the root, the symptoms will worsen and eventually lead to the loss of the teeth [3]. In present times, periodontitis stands as the first cause of tooth loss among adults in worldwide [4].

At present, initial periodontal therapy can effectively control the progression of periodontitis [5]. However, it remains challenging to restore the lost soft and hard tissues damaged by periodontitis. Tissue engineering technology has expected to be used for regenerating periodontal tissue [6]. Seed cells, scaffold materials, and growth factors are the three key elements of the technology [7, 8]. Existed researches have demonstrated that hPDLSCs (human periodontal ligament stem cells) possess the remarkable ability of multipotent differentiation, including osteogenesis, adipogenesis, and fibrogenesis [9–12]. Moreover, hPDLSCs can be easily obtained from the periodontal ligament of premolars that need to be extracted for orthodontic purposes and third molars. Therefore, hPDLSCs represent an ideal choice for periodontal tissue regeneration.

Several cytokines are found in the gingival crevicular fluid of individuals with chronic periodontitis, such as TNF-α (Tumor Necrosis Factor-α), IL-6 and IL-8 [13, 14]. Currently, it is widely accepted that not only plaque biofilm, but also the immune response of the host to the biofilm are two key factors in the development of periodontitis. The plaque biofilm is the primary determinant, while the enzymes, cytokines, and inflammatory mediators produced during the host immune response, that cause damage to the periodontal supporting tissues [15]. Researches have demonstrated that hPDLSCs derived from individuals with periodontitis exhibit significantly reduced osteogenic potential compared to those from periodontally healthy individuals [16, 17]. Numerous inflammatory mediators, such as IL-1, IL-1β, IL-6, IL-11, IL-17, and TNF-α, hinder the process of stem cell osteogenesis. Among them, TNF-α is regarded as the key mediator of the inflammatory response in periodontitis [18]. . By activating various signaling pathways, it inhibits osteogenic differentiation of hPDLSCs, the NF-κB (Nuclear Factor kappa-B) pathway is included [16, 19–22]. Increasingly, researchers believe that anti-inflammatory therapy is just as crucial as antimicrobial therapy in treating periodontitis [23, 24].

The medicinal plant Tripterygium wilfordii is primarily utilized to treat inflammatory and autoimmune diseases, including rheumatoid arthritis, systemic lupus erythematosus, psoriasis, and kidney disease [25–28]. Triptolide, extracted from Tripterygium wilfordii, is one of its main active ingredients, with a molecular formula of C_20_H_24_O_6_ [29]. Triptolide has attracted significant interest in the academic community owing to its strong biological efficacy, significant anti-inflammatory properties, immune regulation capabilities, and its ability to reduce organ transplantation rejection. Previous in vitro studies demonstrated that, via the NF-κB pathway, triptolide reversed the TNF-α-induced inhibition of osteogenic differentiation in C2C12 cells [30]. Animal experiments have demonstrated the potential of triptolide to decelerate orthodontic tooth movement and mitigate root resorption in rats by inhibiting osteoclastogenesis [31]. Additionally, it may have a beneficial impact on osteogenesis. Furthermore, significant anti-inflammatory, anti-osteoclast and pro-osteogenic effects of triptolide have been demonstrated in both in vitro and in vivo studies [32, 33].

The NF-κB pathway comprises two activation pathways: the classical pathway and the non-classical pathway [34]. TNF-α primarily mediates NF-κB signal transduction through the classical pathway [35]. NF-κB is a dimeric complex consisting of p50 and p65 subunits. In the inactive state, NF-κB forms an inactive trimeric complex with its inhibitor IκBα (Inhibitor kappa B alpha) within the cytoplasm. When cells are stimulated by inflammatory factors like TNF-α, activated IκB kinases phosphorylate IκBα, causing its degradation and subsequent release of free NF-κB dimers [36]. These dimers in the cytoplasm are transferred to the nucleus, binding to specific DNA sites and activating downstream target genes. Thus, the IκBα’s phosphorylation and degradation are crucial for NF-κB activation, and inhibiting or reducing IκBα degradation can effectively block the NF-κB pathway. BAY-117,082, a specific NF-κB inhibitor, can impede the IκBα’s phosphorylation and degradation. Inhibition of the NF-κB pathway by BAY-117,082 has been demonstrated to take a turn for the inhibition of TNF-α on osteogenesis and can serve as a positive control in experiments.

The precise impact of triptolide on hPDLSCs within an inflammatory environment and its potential mechanism remain uncertain. Previous research indicates that triptolide exhibits a notable capacity to suppress the phosphorylation of NF-κB, consequently counteracting the inhibition of osteogenic differentiation in osteoblasts induced by TNF-α [30]. Based on these findings, we postulate that triptolide may also counteract the TNF-α-induced inhibition of hPDLSCs’ osteogenic differentiation by modulating the NF-κB pathway. Accordingly, we have devised pertinent experiments to investigate this hypothesis.

## Methods

### Isolation and culture of cell

This study was filed and approved by the Ethics Committee of Shandong University School of Stomatology (No.20,220,506). Premolars from individuals aged 12 to 18 years, requiring extraction for orthodontic reasons, were collected with informed consent from both the children and their parents. Immediately after extraction, the premolars were placed in α-MEM (Hyclone, Logan, UT, USA) containing 5% penicillin/streptomycin that had been pre-cooled to 4 °C. The tube was vigorously shaken to thoroughly remove any blood adhering to the root surface. Within four hours, the teeth were transferred to an ultra-clean workstation, and using PBS to rinse the root surface. Subsequently, scraped the middle half tissue of the root. The tissue block was placed at the bottom wall of the culture flask immediately after scraping. The culture flask was positioned upright, and 5 ml of α-MEM containing 20% FBS (Biological Industries, Israel) was added. After approximately four hours, it was observed that the tissue block had successfully adhered to the bottom of the flask. The flask was then placed flat to allow the medium to be completely free of the tissue block. The old culture medium was replaced by new every three days. After approximately 5–7 days, hPDLSCs could be observed migrating out from the edges of the tissue block. Around two weeks later, the hPDLSCs had grown to cover approximately 80% of the flask’s bottom surface. The hPDLSCs were digested with 1 ml of trypsin-EDTA solution (Yeasen, Shanghai, China). After about 1.5 min, the hPDLSCs could be observed detaching and assuming a round, spherical shape under the microscope. The digestion was halted by adding 3 ml of complete medium containing 10% FBS. The hPDLSCs were then resuspended in complete medium and placed in larger culture dishes for continued growth.

### Flow cytometry

HPDLSCs in the logarithmic growth phase were subjected to digestion, followed by centrifugation. Subsequently, the cells were resuspended in PBS again to achieve a cell density of 1 × 10^7^ cells per ml. Five 1.5 ml EP tubes were prepared, and added 100 µl cell suspension to each EP tube. In a dark environment, the appropriate amount of CD34, CD44, CD45, and CD105 antibodies (Elabscience, Houston, TX, USA) was added to each EP tube, while the control tube received the same volume of PBS. After 20 min of incubating on ice, the cells were then subjected to two additional washes. Finally, the hPDLSCs were resuspended using 500 µl of PBS, filtered through a nylon mesh, and loaded into flow tubes for analysis using flow cytometry.

### Cell proliferation assay

The hPDLSCs were routinely digested and resuspended through centrifugation. Subsequently, cells were cultured in 96-well plates for 24 h, different stimuli were added. Each concentration had five replicate wells. On days 1, 3, and 5, following the washes, the experimental wells were supplemented with CCK-8 solution. After incubation, using the microplate reader to measure the optical density values at OD450 nm.

### Oil Red O staining

HPDLSCs were added to 6-well plate at a density of 100,000 cells per well. 1 day later, added the adipogenic induction medium (α-MEM contains 10% FBS, 10 mM insulin, 0.5 M hydrocorti-sone, 500mM isobutyl-methylxanthine and 60mM indomethacin). After 28-day of adipogenic induction culture, the old medium was discarded. Next, used 4% paraformaldehyde to fix cells, followed by another wash with PBS. Oil Red O staining solution (Beyotime, Shanghai, China) was used to observe whether lipid droplets were formed.

### Alizarin red staining and calcium quantitative assay

In this experiment, the same cell culture method as the last experiment was used. However, instead of adipogenic induction medium, osteogenic induction medium (α-MEM contains 10% FBS, 0.01µM dexamethasone, 10mM β-glycerophosphate and 50 µg/mL ascorbic acid) was used. HPDLSCs with three different concentrations of stimulation were cultured for 28 days: blank control, 20ng/ml TNF-α, and 20ng/ml TNF-α + 10^− 11^M triptolide (Solarbio, Beijing, China). After the 28-day period of osteogenic induction, the alizarin red staining solution (OriCell, Guangzhou, China) was employed to stain the mineralised nodules.

Following the staining, the cells were subjected to gross and microscopic observation. To fully dissolve the mineralized nodules, 10% cetylpyridinium chloride was added. The OD562 nm was then measured using the microplate reader.

### ALP staining and activity assay

HPDLSCs were added at a density of 200,000 cells per well to 6-well plates. Concurrently with osteogenic induction culture, hPDLSCs were subjected to a 7-day treatment with culture media supplemented with or without TNF-α and/or triptolide. After the 7-day culture period, qualitative observations were detected by ALP staining kit (Jiancheng, Nanjing, China). Darker blue staining indicated stronger ALP activity, which is indicative of osteogenic differentiation.

In a separate set of wells, hPDLSCs were cultured in the same manner as described above. On day 7, the hPDLSCs were washed with ice-cold PBS. Next, cell lysate (RIPA buffer: PMSF = 99: 1) was added at 200 µl to each well, and the hPDLSCs were lysed for 15 min on ice. After centrifugation at low temperature and high speed, carefully aspirated the supernatant and transferred to EP tube.

The BCA method was used to quantify the concentration of extracted protein. Finally, a small amount of total protein was taken to detecte the ALP activity.

### Reverse transcription-quantitative PCR (RT-qPCR)

After 7-day of osteogenic induction culture, Trizol method was used to extract total RNA of the cells. The extracted RNA was reverse transcribed into complementary DNA (cDNA) using the reverse transcription kit (Yeasen, Shanghai, China).

Each reaction system consisted of three replicate wells. By designed and analyzed, in this experiment, the primer sequences were as follows:

-IL-6: 5’-ATAACCACCCCTGACCCAAC-3’ and 5’-CCCATGCTACATTTGCCGAA-3’;
-IL-8: 5’-TCAGAGACAGCAGAGCACAC-3’ and 5’-GGCAAATGCACTTTCACACA-3’;
-ALP: 5’-GGCGGTGAACGAGAGAATGT-3’ and 5’-GGACGTAGTTCTGCTCGTGG-3’;
-RUNX2: 5’-GGAGTGGACGAGGCAAGAGT-3’ and 5’-AGGCGGTCAGAGAACAAACT-3’;
-COL1: 5’-TAAAGGGTCACCGTGGCTTC-3’ and 5’-GGGAGACCGTTGAGTCCATC-3’;
-GAPDH: 5’-GCACCGTCAAGGCTGAGAAC-3’ and 5’-TGGTGAAGACGCCAGTGGA-3’.

The 2^−ΔΔCt^ method was used to analyse the data obtained, which allows for the relative quantification of gene expression.

### Western blotting

It was used the protein samples, with a guaranteed amount of 20 micrograms of protein in each loading well. Then, 10% SDS-PAGE was used to separate the target proteins with different molecular weights. Following the transfer of the target protein onto a PVDF membrane, the membrane was blocked 2 h using 5% non-fat milk solution to minimize non-specific antibody binding. After thorough washing with TBST solution, using primary antibodies of target proteins (ALP, RUNX2, COL1, GAPDH) to incubate the membrane overnight at 4 °C, according to their respective molecular weights. After another round of washing with TBST solution to remove unbound primary antibodies, using secondary antibodies to incubate the membrane for 1 h. Finally, washing the membrane again to remove any residual antibodies, and the protein bands were exposed using a chemical hypersensitive luminescent liquid kit (Biosharp, Beijing, China).

In this study, for ease of operation, the blots were cut prior to hybridisation with antibodies during blotting.

### NF-kB pathway studies

BAY-117,082 is a precise inhibitor of the NF-κB pathway, effectively impeding the phosphorylation of IκBα and consequently hindering the NF-κB pathway. In this particular experiment, BAY-117,082 was employed as the positive control. The hPDLSCs were cultivated in six-well plates and underwent a 7-day osteogenic induction. Subsequently, they were pre-treated with either BAY-117,082 or triptolide for 1 h. Finally, TNF-α was introduced to activate the NF-κB pathway. Then proteins within the hPDLSCs were isolated. To detect the presence of proteins associated with the NF-κB pathway (NF-κB, p-NF-κB, and p-IκBα), Western blot analysis was performed.

### Statistical analysis

The experiments were performed at least 3 times, independent experiments for each repeat in this study. Analysis was performed using SPSS 26.0 software. The mean ± standard deviation represents the experimental data. The statistical significance has been established at a level of significance of *P* < 0.05. Subsequently, the results of the data analysis were graphically presented using GraphPad Prism 9.0.

## Results

### Isolation, cultivation and characterization of hPDLSCs

**Fig. 1:**
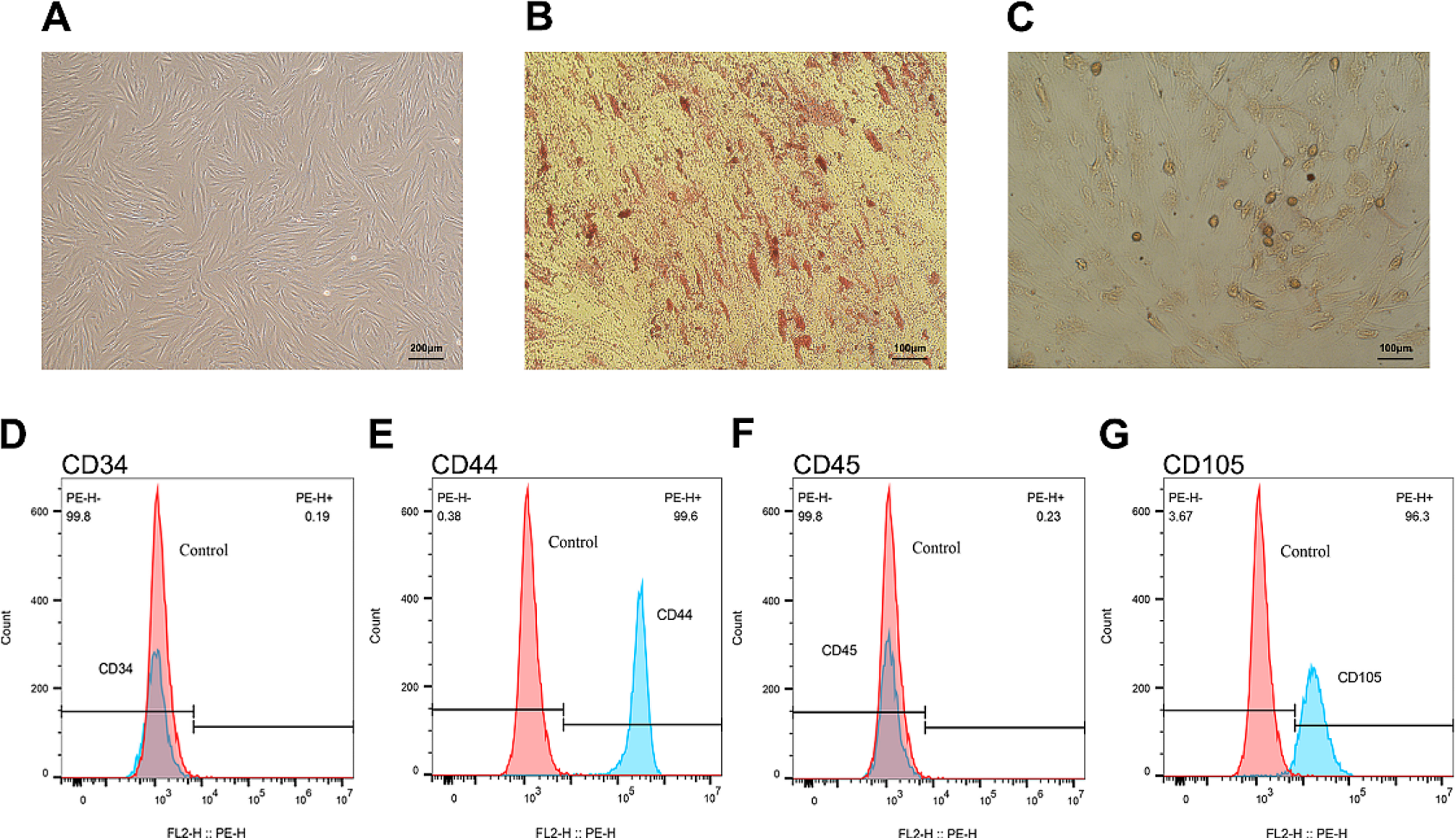
Isolation, cultivation and characterization of hPDLSCs. (**A**) hPDLSCs showed a long spindle shape and arranged in feathers. Scale bar: 200 μm. (**B**) After osteogenic differentiation induction, mineralized nodules were observed. Scale bar: 100 μm. (**C**) After adipogenic differentiation induction, lipid droplets were observed. Scale bar: 100 μm. (**D, E, F, G**) CD34, CD44, CD45 and CD105 expression were determined. CD44 (**E**) and CD105 (**G**) were high expression, while the CD34 (**D**) and CD45 (**F**) were low expression

After about 1 to 2 weeks, at the edge of the periodontal tissue block, primary cells could be observed crawling out. We took the primary cells for digestion culture, and used the 3–5 generation cells for follow-up experiments (Fig. 1A). After 21 days of osteogenic induction culture and 28 days of lipogenic induction culture, hPDLSCs were stained with Alizarin Red and Oil Red O, which showed that the cells had the ability of multiple differentiation (Fig. 1B and C). Flow cytometry demonstrated pessimistic expression of CD34 and CD45 antibodies and affirmative expression of CD44 and CD105 antibodies on the exterior of the cell, indicating that the isolated cells were stem cells (Fig. 1D, E, F, G).

### Proliferation of hPDLSCs following treatment with triptolide and TNF-α

**Fig. 2:**
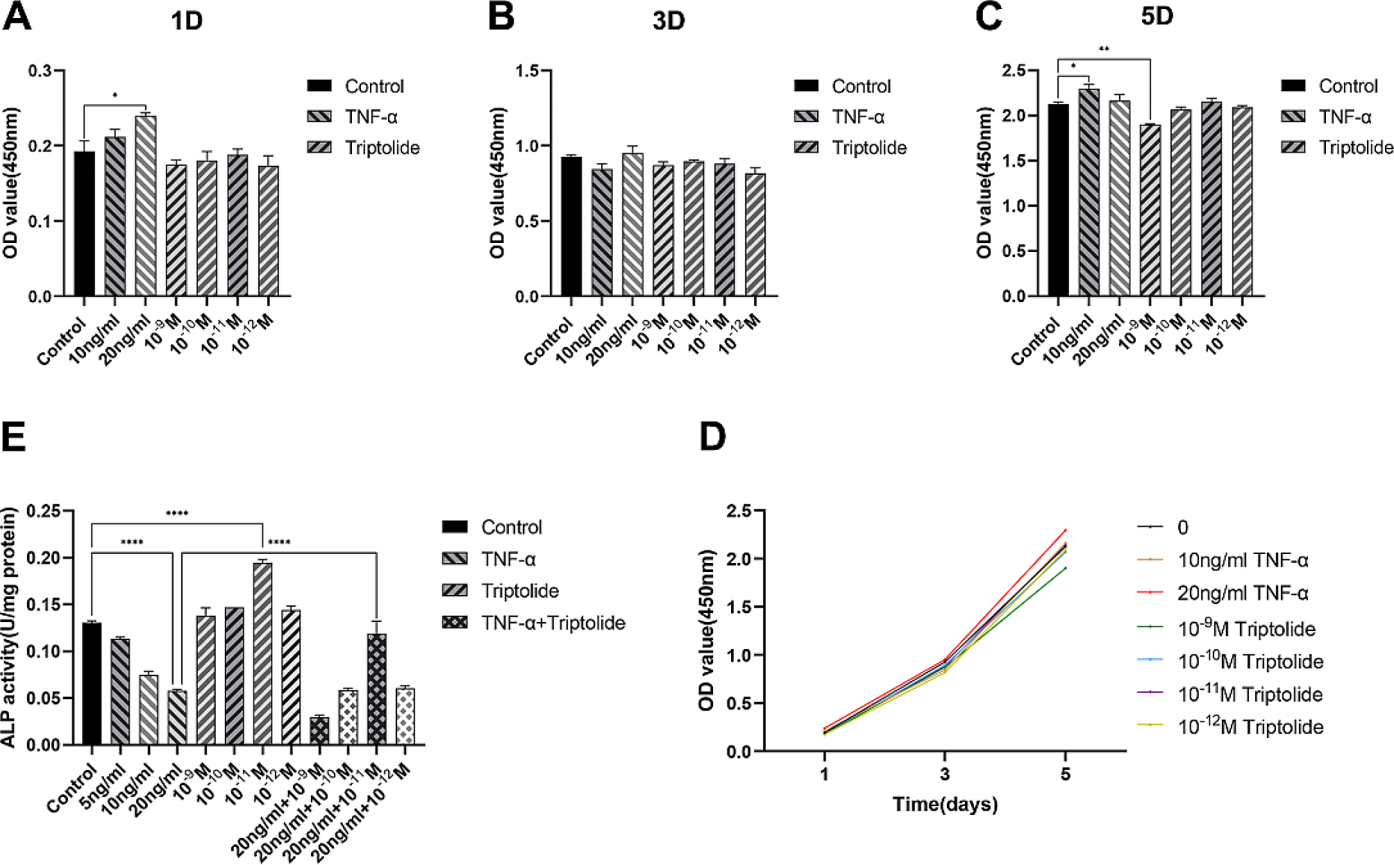
The determination of TNF-α and triptolide concentrations. (A, B, C) CCK-8 assay demonstrated the proliferation of hPDLSCs treated for 1 (**A**), 3 (**B**), 5 (**C**) days with control, TNF-α (10, 20ng/ml) or triptolide (10^− 9^M, 10^− 10^M, 10^− 11^M, 10^− 12^M). (**D**) The growth curves of the control, TNF-α or triptolide treated at different concentration on day 1, 3, 5. (**E**) ALP activity under control, TNF-α (5, 10, 20ng/ml) or/and triptolide (10^− 9^M, 10^− 10^M, 10^− 11^M, 10^− 12^M) treatment

The CCK-8 assay was used to evaluate the proliferation of hPDLSCs exposed to TNF-α and triptolide for 1, 3 and 5 days. The experiment showed that 20ng/ml TNF-α on day 1 (Fig. 2A) and 10ng/ml TNF-α on day 5 (Fig. 2C) could promote cell proliferation, but it was not obvious. On day 3, different concentrations of TNF-α or triptolide had no significant effect on cell proliferation (Fig. 2B). On day 5, 10^− 9^M triptolide had a slight inhibitory effect on cell proliferation (Fig. 2C). The hPDLSCs were exposed to various concentrations of TNF-α and triptolide gradually increased in proliferative capacity from day 1 to day 5 (Fig. 2D).

### Effects of different doses of triptolide and TNF-α on hPDLSCs osteogenesis

ALP activity experiment indicated that different levels of TNF-α dose-dependently inhibited hPDLSC osteogenesis (Fig. 2E), and 20ng/ml TNF-α inhibition was the most significant. However, different concentrations of triptolide could promote the osteogenesis of hPDLSCs, among which 10^− 11^M had the most significant effect and could reverse the inhibition of the activity of ALP that was induced by TNF-α. Therefore, subsequent experiments were carried out at concentration of 20ng/ml TNF-α and/or 10^− 11^M triptolide.

### Triptolide restores osteogenesis in hPDLSCs inhibited by TNF-α

**Fig. 3:**
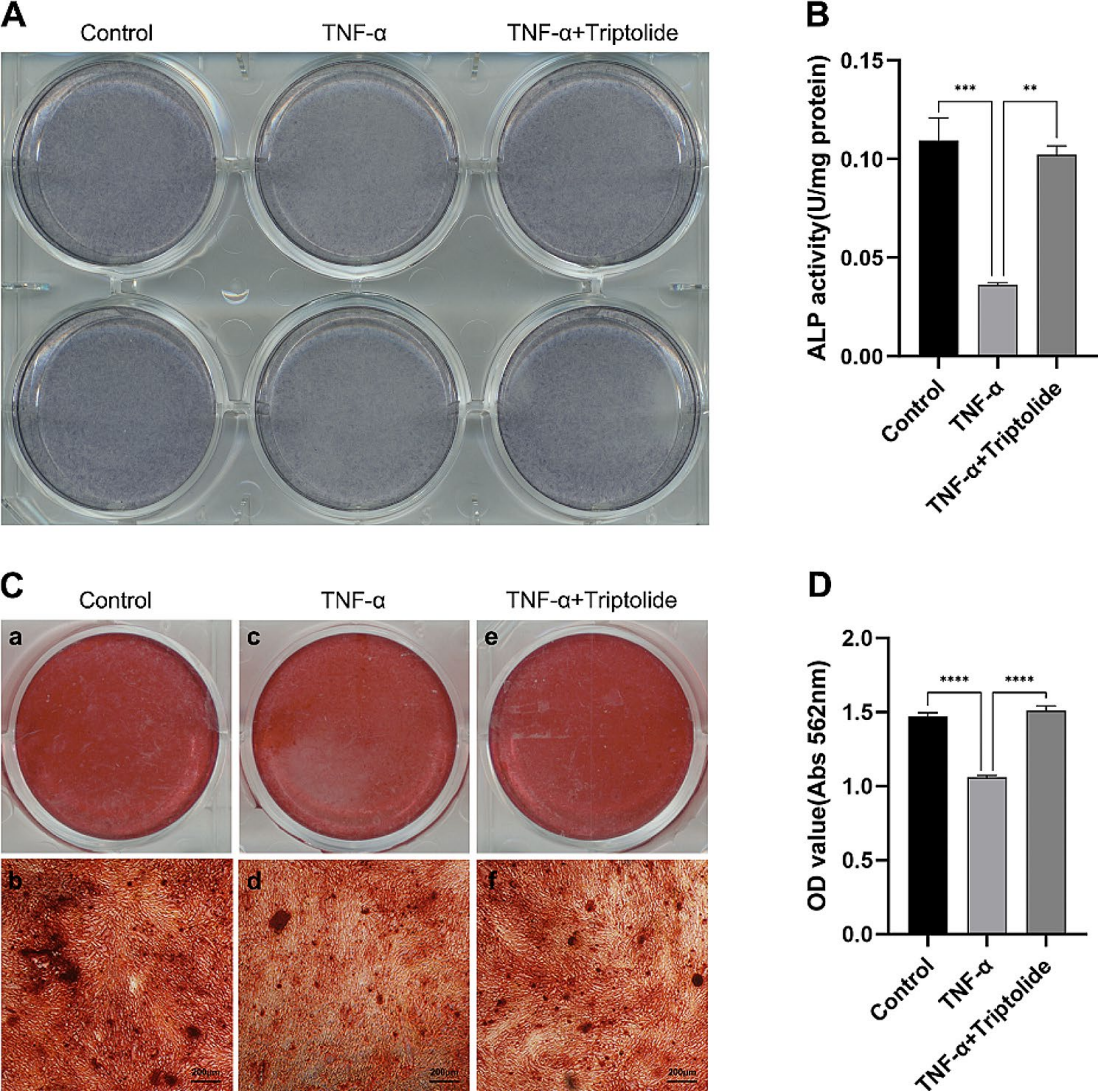
Effects of TNF-α and triptolide on ALP activity and mineralized nodule deposition of hPDLSCs. (**A**) ALP staining under control, TNF-α, TNF-α (20ng/ml) and triptolide (10^− 11^M) treatment after osteogenic induction for 7 days. (**B**) ALP activity under control, TNF-α, TNF-α (20ng/ml) and triptolide (10^− 11^M) treatment for 7 days. (**C**) Formation of mineralized nodules under control (ab), TNF-α (cd), TNF-α (20ng/ml) and triptolide (10^− 11^M) (ef) treatment for 21 days. (**D**) Extracellular matrix mineralisation was assessed at OD562

ALP staining showed that hPDLSCs staining degree of TNF-α stimulation was lighter than that of control group and TNF-α + triptolide group, however, the staining degree of the latter two groups was similar (Fig. 3A). Triptolide had been shown to be a reversal of TNF-α-induced inhibition of osteogenesis in hPDLSCs. The quantitative experiments of ALP activity showed similar results (Fig. 3B).

The number and size of mineralised nodules were smaller in the TNF-α-treated group than in the control and TNF-α + triptolide groups, as shown by Alizarin red staining (Fig. 3C and D).

### Triptolide reverses TNF-α-induced decrease in the expression of osteogenesis-related m-RNA and proteins in hPDLSCs

**Fig. 4:**
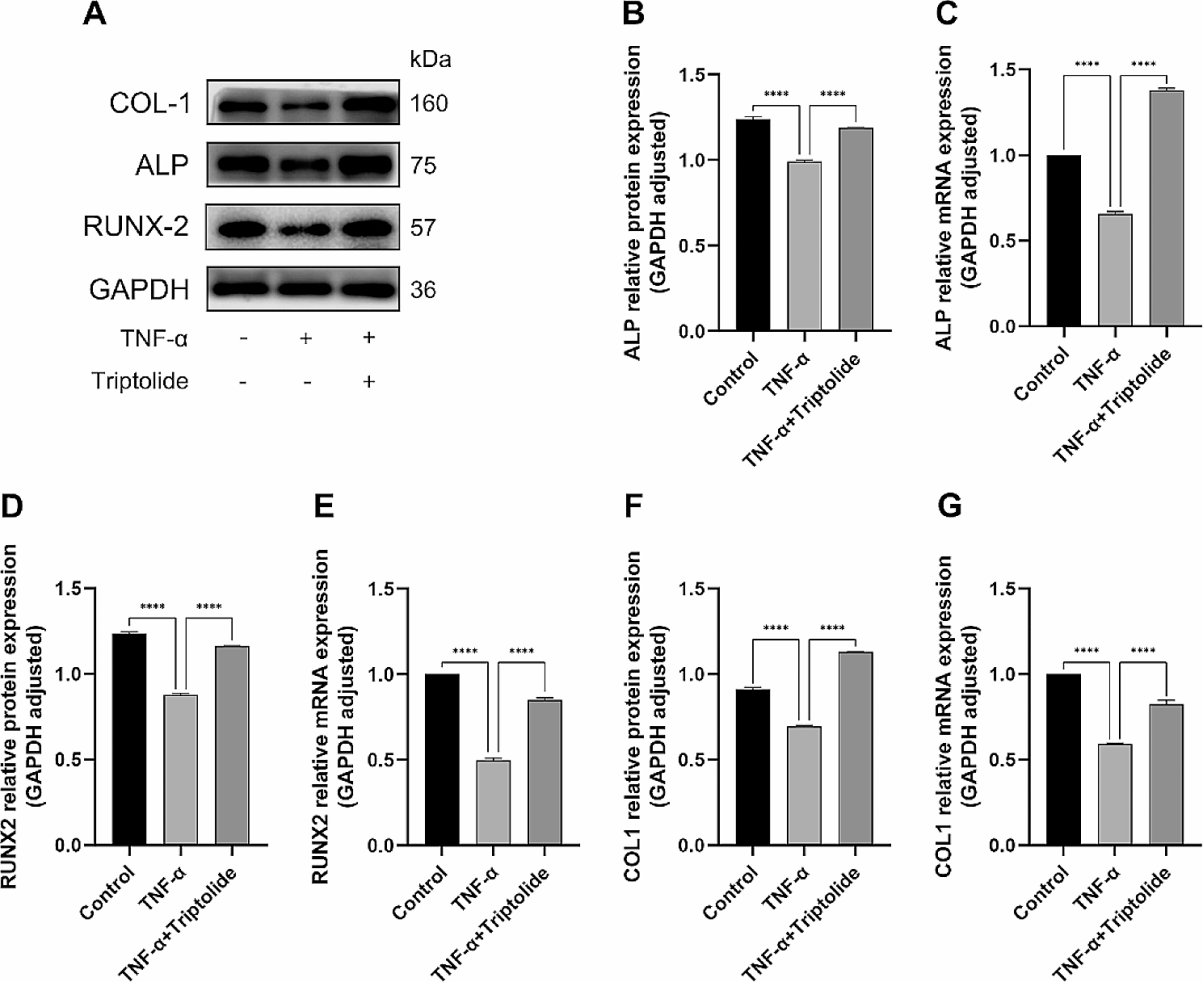
Triptolide rescues TNF-α-induced inhibition of osteogenesis in hPDLSCs. (**A**) Western blot assay for ALP, RUNX2 and COL1 protein expression in hPDLSCs after osteogenic induction for 7 days under control, TNF-α, TNF-α (20ng/ml) and triptolide (10^− 11^M) treatment. (**B**) Semi-quantitative analysis of ALP protein levels in hPDLSCs. (**C**) Relative expression of ALP mRNA. (**D**) Semi-quantitative analysis of RUNX2 protein levels in hPDLSCs. (**E**) Relative expression of RUNX2 mRNA. (**F**) Semi-quantitative analysis of COL1 protein levels in hPDLSCs. (**G**) Relative expression of COL1 mRNA. The blots were cut prior to hybridisation with antibodies during blotting

After 7 days of osteogenic induction, the expression of osteogenesis-associated genes ALP, RUNX2, and COL1 m-RNA was detected in hPDLSCs. (Fig. 4C, E, G). In those treated with TNF-α, related genes were found to become downregulated. In those treated with TNF-α + triptolide, related genes were found to become upregulated. Subsequently, a similar trend was observed in the western blot assay of the osteogenesis-related proteins ALP, RUNX2 and COL1 (Fig. 4A), and the gray value analysis of their bands showed statistically significant differences (Fig. 4B, D, F).

### Triptolide reduces the expression of inflammatory cytokines induced by TNF-α in hPDLSC

**Fig. 5:**
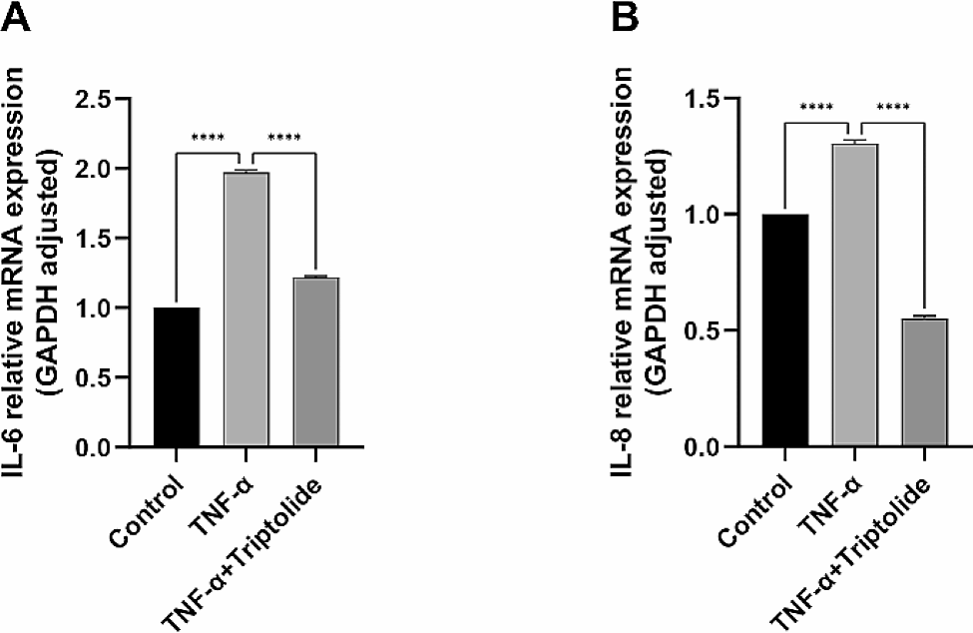
Triptolide reduces the level of inflammatory factors induced by TNF-α in hPDLSCs. (**A**) Relative IL-6 mRNA expression in hPDLSCs after osteogenic induction for 7 days under control, TNF-α, TNF-α (20ng/ml) and triptolide (10^− 11^M) treatment. (**B**) Relative expression of IL-8 mRNA in hPDLSCs

To examine the anti-inflammatory ability of triptolide on TNF-α-treated hPDLSCs, the m-RNA expression of IL-6 and IL-8 were also examined (Fig. 5A and B). After TNF-α treatment, the RT-qPCR results indicated a significant increase in IL-6 and IL-8 m-RNA expression. However, the m-RNA expression level of IL-6 and IL-8 in the TNF-α + Triptolide group was significantly lower than that in the group treated with TNF-α alone.

### Determination of the activation time of the NF-κB pathway

**Fig. 6:**
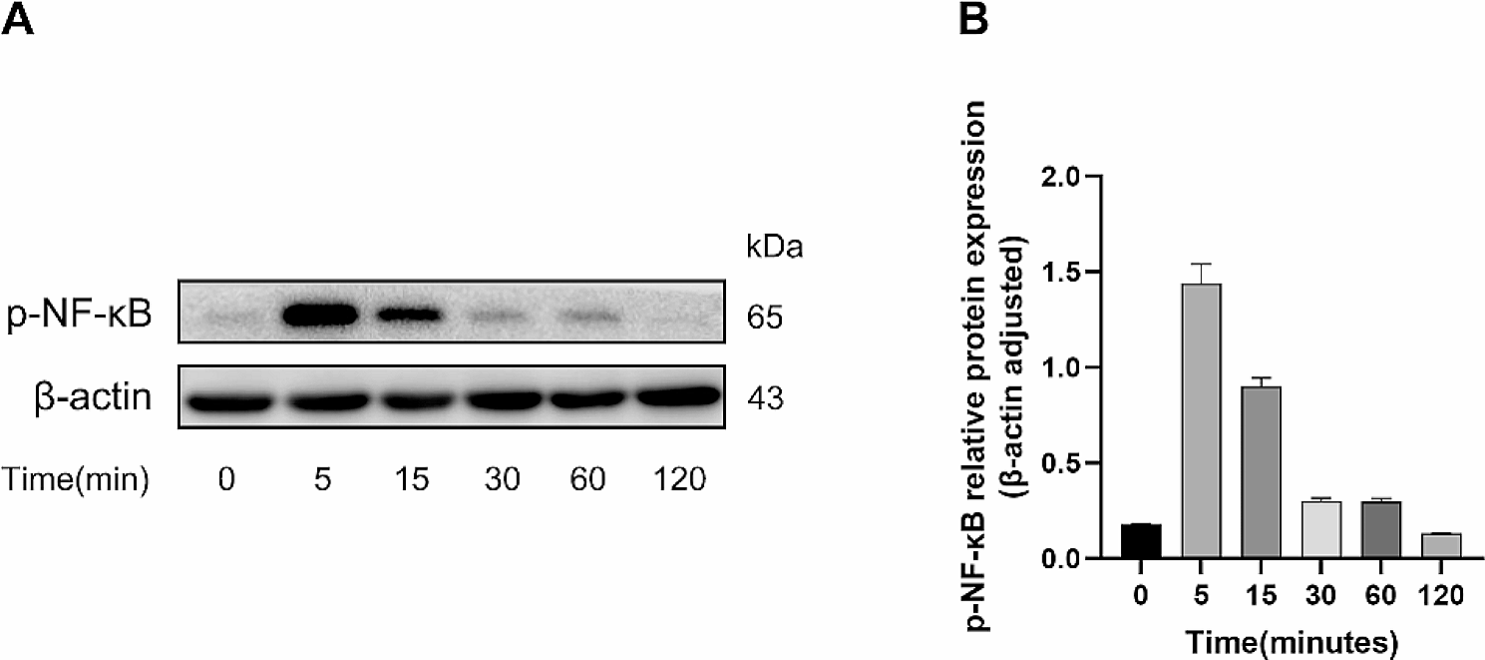
Activation of the NF-κB pathway after addition of TNF-α at different time points. (**A**) Western blot assay for p-NF-κB protein expression in hPDLSCs after osteogenic induction for 7 days under TNF-α (20ng/ml) treatment. (**B**) Semi-quantitative analysis of p-NF-κB protein levels in hPDLSCs. The blots were cut prior to hybridisation with antibodies during blotting

In order to determine the most appropriate time point for the activation of NF-κB pathway after adding TNF-α, we selected 6 time points of 0 min, 5 min, 15 min, 30 min, 60 min, 120 min to detect p-NF-κB protein expression (Fig. 6A and B). The experimental results suggest that p-NF-κB protein expression was the highest at 5 min after adding TNF-α, and then decreased gradually as time went on. Therefore, the time node of adding TNF-α 5 min was used for the subsequent detection of p-NF-κB pathway-related proteins.

### Triptolide rescues the inhibition of osteogenesis induced by TNF-α via blocking the NF-κB pathway

**Fig. 7:**
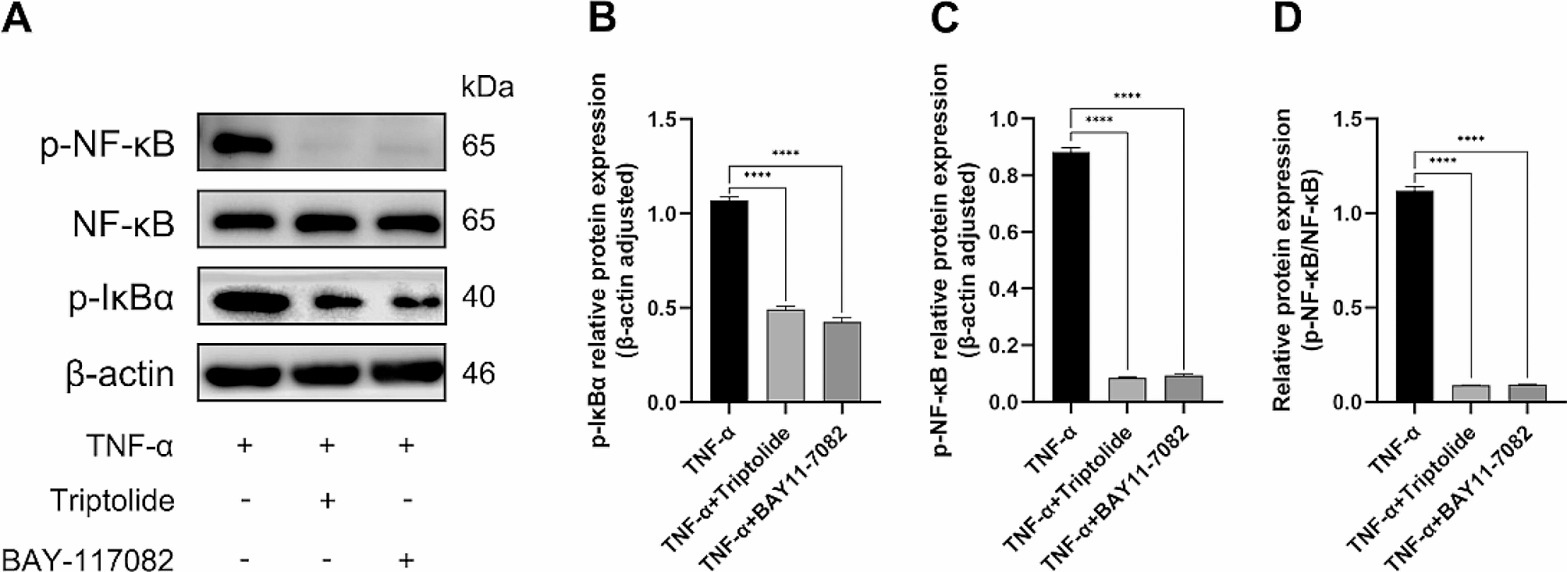
Triptolide rescues TNF-α-induced inhibition of osteogenesis in hPDLSCs via the p-IκBα/NF-κB pathway. (**A**) p-IκBα, NF-κB and p-NF-κB protein expression in hPDLSCs after osteogenic induction for 7 days under TNF-α, TNF-α (20ng/ml) and triptolide (10^− 11^M), TNF-α (20ng/ml) and BAY11-7082 (10^− 5^M) treatment. (**B**) Semi-quantitative analysis of p-IκBα protein levels in hPDLSCs. (**C**) Semi-quantitative analysis of p-NF-κB protein levels in hPDLSCs. (**D**) Relative p-NF-κB/NF-κB protein expression levels. The blots were cut prior to hybridisation with antibodies during blotting

For further investigation into the potential mechanism by which triptolide counteracts the TNF-α-induced suppression of osteogenesis in hPDLSCs. BAY-117,082, a pathway specific inhibitor of NF-κB pathway, was used in the experiment. HPDLSCs that had been osteogenic for 7 days were pre-treated with triptolide (10^− 11^M) or BAY-117,082 (10^− 5^M) for 1 h, and then treated with TNF-α (20ng/ml) for 5 min. Finally, proteins were extracted from the hPDLSCs for Western blot assay. From the experimental results, triptolide and BAY-117,082 treatment groups showed the same results, that is, p-NF-κB and p-IκBα protein expression was significantly lower than that of TNF-α-only treatment group (Fig. 7A). Moreover, the protein bands were analyzed using gray-scale methods, which revealed that there was a substantial decrease in the expression of p-NF-κB/ NF-κB protein after triptolide and BAY-117,082 treatment compared to TNF-α treatment alone (Fig. 7B, C, D).

## Discussion

The repair of periodontal supporting tissue defects caused by periodontitis is a challenging and widely-discussed issue. In the field of periodontitis treatment, it is commonly accepted among scholars that periodontal surgery should be performed to repair the damaged periodontal soft and hard tissues, in conjunction with effective plaque control [37]. In recent years, the advancements in tissue engineering technology have provided a promising solution for the restoration of both congenital and acquired human soft and hard tissue defects [38].

HPDLSC were initially discovered by Seo et al. in 2004 [39]. These cells possess the remarkable ability of multilineage differentiation, including osteoblasts, adipocytes, fibroblasts, etc. Considering its multi-directional differentiation potential, hPDLSCs have been thought as an ideal choice for periodontal tissue regeneration [40, 41]. Moreover, hPDLSCs can be easily obtained from extracted premolars and wisdom teeth.

Previous studies mainly focused on the pathogenic factors such as dental plaque and lipopolysaccharide [42, 43], because it was generally believed that pathogenic factors caused the destruction of periodontal tissue in the past. However, it’s difficult to explain why patients with the same plaque level show different degrees of periodontal tissue destruction. Further research has revealed a positive correlation between the levels of inflammatory factors in the periodontal crevicular fluid of periodontitis patients and the extent of periodontal tissue damage. Consequently, an increasing number of scholars now believe that periodontitis is triggered by dental plaque, and the subsequent immune response by the host leads to the generation of inflammatory factors, that ultimately leads to the destruction of periodontal supporting tissues [44]. That is to say that the role of the host’s inflammatory immune response is crucial to both the tissue destruction and regeneration associated with periodontitis.

TNF-α plays an important role among the various inflammatory factors associated with periodontitis. TNF-α exhibits the ability to activate multiple signaling pathways, contributing to the destroying periodontal tissue and inhibiting new bone formation. In all of these signaling pathways activated by TNF-α, the NF-κB pathway stands out as the most critical one. By regulating genes expression, NF-κB participates in a range of biological processes, including immune response, inflammatory response, cell apoptosis, and tumorigenesis, etc [45]. Moreover, TNF-α has been observed to inhibit the Wnt pathway and BMP-Smads pathway by activating the NF-κB pathway [46–48]. This has a negative implications to the osteogenic differentiation of stem cells. Consequently, in this research, TNF-α was employed as a proinflammatory factor, and the NF-κB pathway was specifically selected as the subject of investigation.

Some researchers have discovered that even after undergoing systematic periodontal initial treatment, patients with periodontitis still exhibit a certain concentrations of inflammatory factors in their gingival crevicular fluid [49]. This suggests that in addition to the standard periodontal treatment and periodontal tissue regeneration therapy, simultaneous anti-inflammatory treatment should be implemented. This approach can be effective in the inhibition of further periodontal tissue loss and the promotion of new periodontal tissue regeneration.

Tripterygium wilfordii, a widely used traditional Chinese medicine, has gained attention in this context. One of its primary active components is triptolide. Triptolide is a natural product with multiple biological activities, derived from Tripterygium wilfordii. Numerous studies have highlighted the anti-inflammatory, anti-rheumatic, anti-cancer, and anti-Alzheimer’s disease effects of triptolide [50, 51]. Given these properties, we have chosen triptolide as the subject of our research and designed relevant experiments to investigate its impact on the osteogenesis of hPDLSCs in an inflammatory environment.

We used the tissue block method to acquire primary hPDLSCs. Subsequently, hPDLSCs from passages 3 to 5 were utilized for the experimental procedures. Following induction of osteogenesis and adipogenesis, we verified the multi-directional differentiation potential of the obtained cells. Mesenchymal stem cell characteristics were confirmed through flow cytometry analysis of the isolated cells. Based on these findings, we identified the isolated cells as hPDLSCs and proceeded with further experiments.

The CCK-8 assay demonstrated that various TNF-α and triptolide concentrations do not significantly impact hPDLSC proliferation. Conversely, TNF-α significantly inhibited the osteogenic capacity of hPDLSCs in the ALP experiment, particularly at a concentration of 20ng/ml. Additionally, 10^− 11^M triptolide alone exhibited a remarkable promotion effect on osteogenesis. The combined treatment of 20ng/ml TNF-α and 10^− 11^M triptolide effectively counteracted the osteogenesis. Therefore, we selected 20ng/ml TNF-α and 10^− 11^M triptolide for subsequent experiments.

At the cellular level, various assays including ALP staining, ALP quantification, alizarin red staining, and calcium quantification revealed a significant suppression of osteogenesis in hPDLSCs by TNF-α. However, the addition of triptolide reversed the suppression effect. At the molecular level, osteogenesis-associated genes and proteins were examined, such as ALP, RUNX-2, and COL-1. The outcomes revealed that TNF-α inhibited their expression, whereas triptolide reversed the suppression. Moreover, IL-6 and IL-8 gene expression was increased by TNF-α, while triptolide reduced them. These results suggest that triptolide is capable of counteracting the suppressive effects of TNF-α on osteogenesis in hPDLSCs.

In the last part of this study, our focus turned to the NF-κB pathway. BAY-117,082 is the specific inhibitor of the NF-κB pathway, through preventing the phosphorylation and degradation of IκBα. In this study, we used BAY-117,082 as the positive control. The findings indicate that the NF-κB pathway was promptly activated upon the introduction of TNF-α in hPDLSCs. The concentration of p-NF-κB protein reached its maximum at the 5 min and then gradually decreased, approaching negligible levels at the 120 min. However, triptolide exhibited the same effect as BAY-117,082 in inhibiting the production of p-IκBα and p-NF-κB proteins. That means by inhibiting the phosphorylation of IκBα and the subsequent release of NF-κB dimers, triptolide may inhibit the activation of the NF-κB pathway. While the present study has demonstrated the promising anti-inflammatory and osteopromotive effects of triptolide, it is important to consider its narrow therapeutic window and potential for multi-organ toxicity when evaluating its clinical application. It is worth noting that the use of protective agents, identification of the toxic dose range, and implementation of a toxicity warning system can help mitigate the negative effects of triptolide [52]. In the future, triptolide may be an excellent drug for treating chronic periodontitis and promoting bone regeneration. More experiments, including vivo experiments, need to be conducted, which will be the focus of our future research.

## Conclusion

In conclusion, by inhibiting the NF-κB pathway, triptolide (10^− 11^M) exhibited the capacity to counteract the suppressive effects of TNF-α-induced (20ng/ml) osteogenesis in hPDLSCs.

### Electronic supplementary material

Below is the link to the electronic supplementary material.

Supplementary Material 1

## Data Availability

The datasets during and/or analysed during the current study available from the corresponding author on reasonable request.

## Abbreviations

hPDLSCs: Human periodontal ligament stem cells
TNF-α: Tumor necrosis factor-α
IL: Interleukin
NF-κB: Nuclear factor kappa-B
IκBα: Inhibitor kappa B alpha
PBS: Phosphate-buffered Saline
α-MEM: α-minimum essential medium
FBS: Fetal bovine serum
CCK-8: Cell counting kit-8
ALP: Alkaline phosphatase
RUNX2: RUNX Family Transcription Factor 2
COL1: Collagen type I
BCA: Bicinchoninic acid assay
GAPDH: Glyceraldehyde-3-phosphate dehydrogenase
β-actin: Beta cytoskeletal actin
CPC: Cetylpyridinium chloride
SDS-PAGE: Sodium dodecyl sulfate-polyacrylamide gel electrophoresis
PVDF: Polyvinylidene fluoride
p-NF-κB: Phosphonated Nuclear Factor-Kappa B
p-IκBα: Phosphonated Inhibitor kappa B alpha
