# Supplementary figures and images for: Triptolide mitigates the inhibition of osteogenesis induced by TNF-α in human periodontal ligament stem cells via the p-IκBα/NF-κB signaling pathway: an in-vitro study
Source: BMC Complement Med Ther. 2024 Mar 6;24:113. doi: 10.1186/s12906-024-04408-2 (PMC10916329; doi:10.1186/s12906-024-04408-2)

Figure 4A

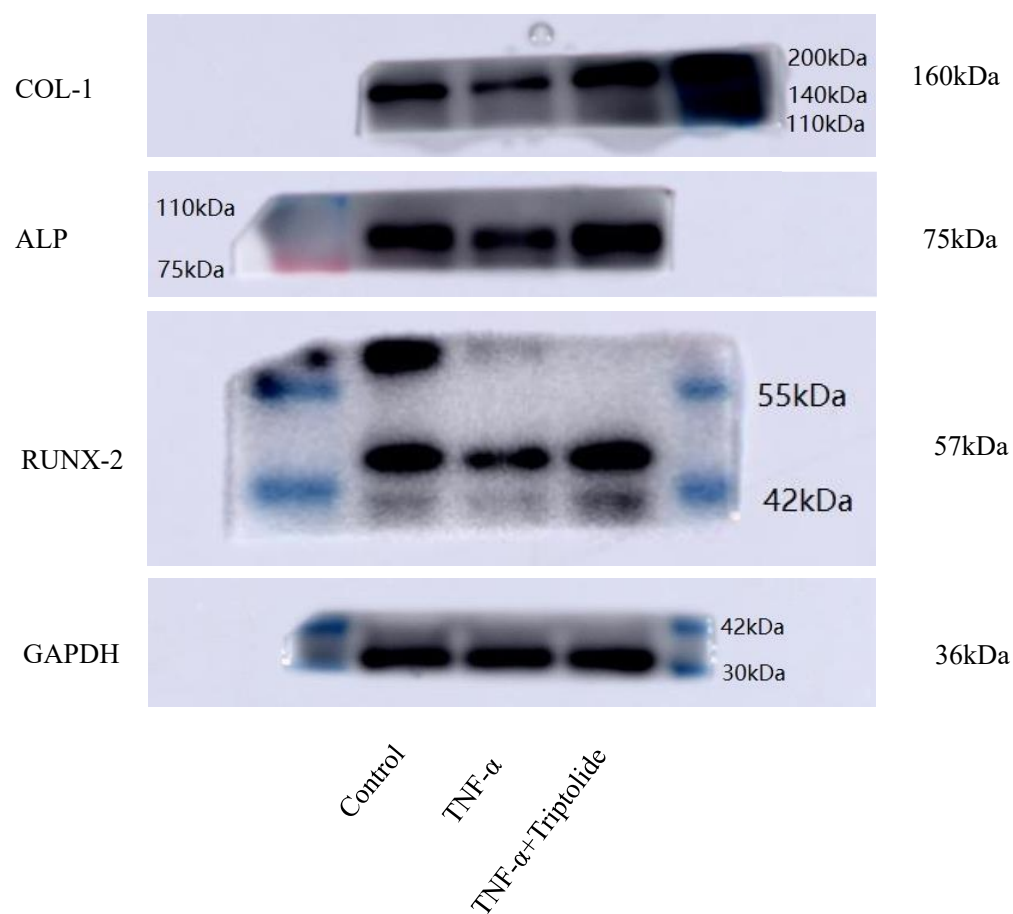

Figure 6A

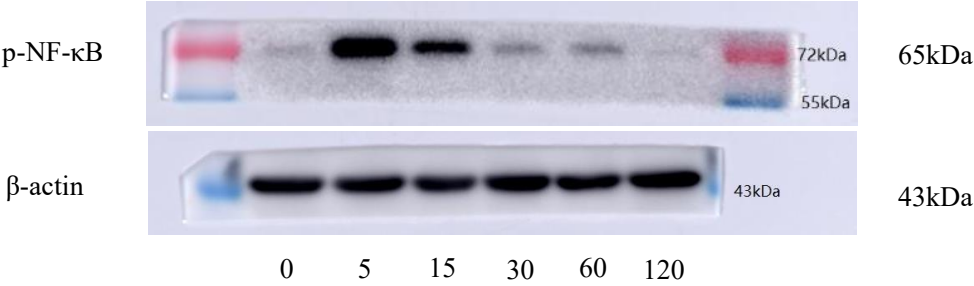

Figure 7A

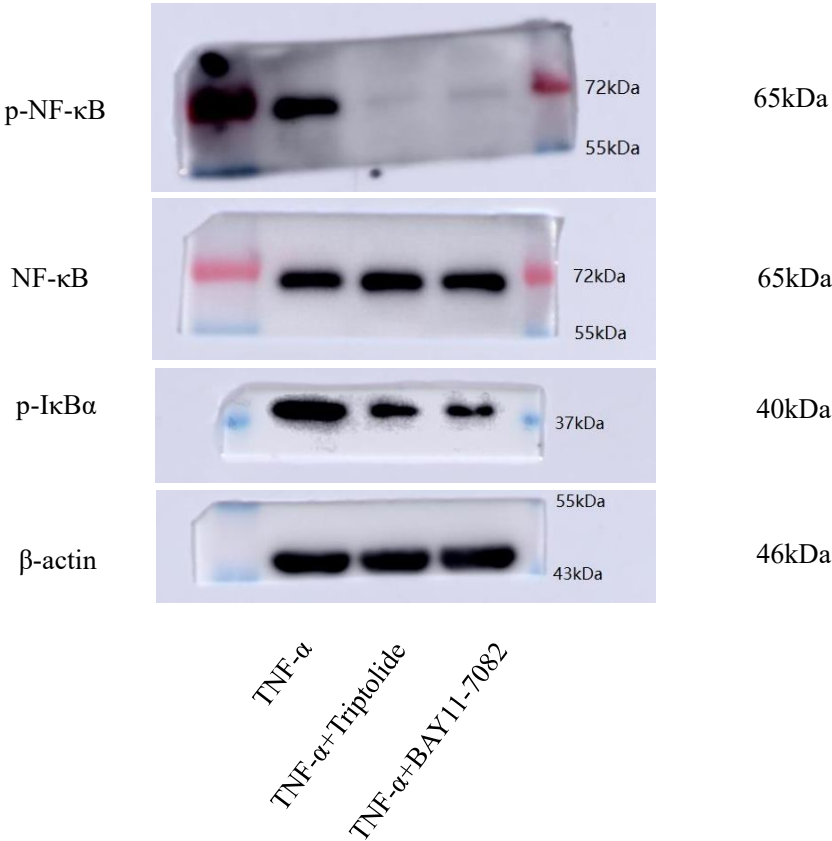

Supplement: Supplementary file 1 — Supplementary Material 1 [file 12906_2024_4408_MOESM1_ESM.pdf]
